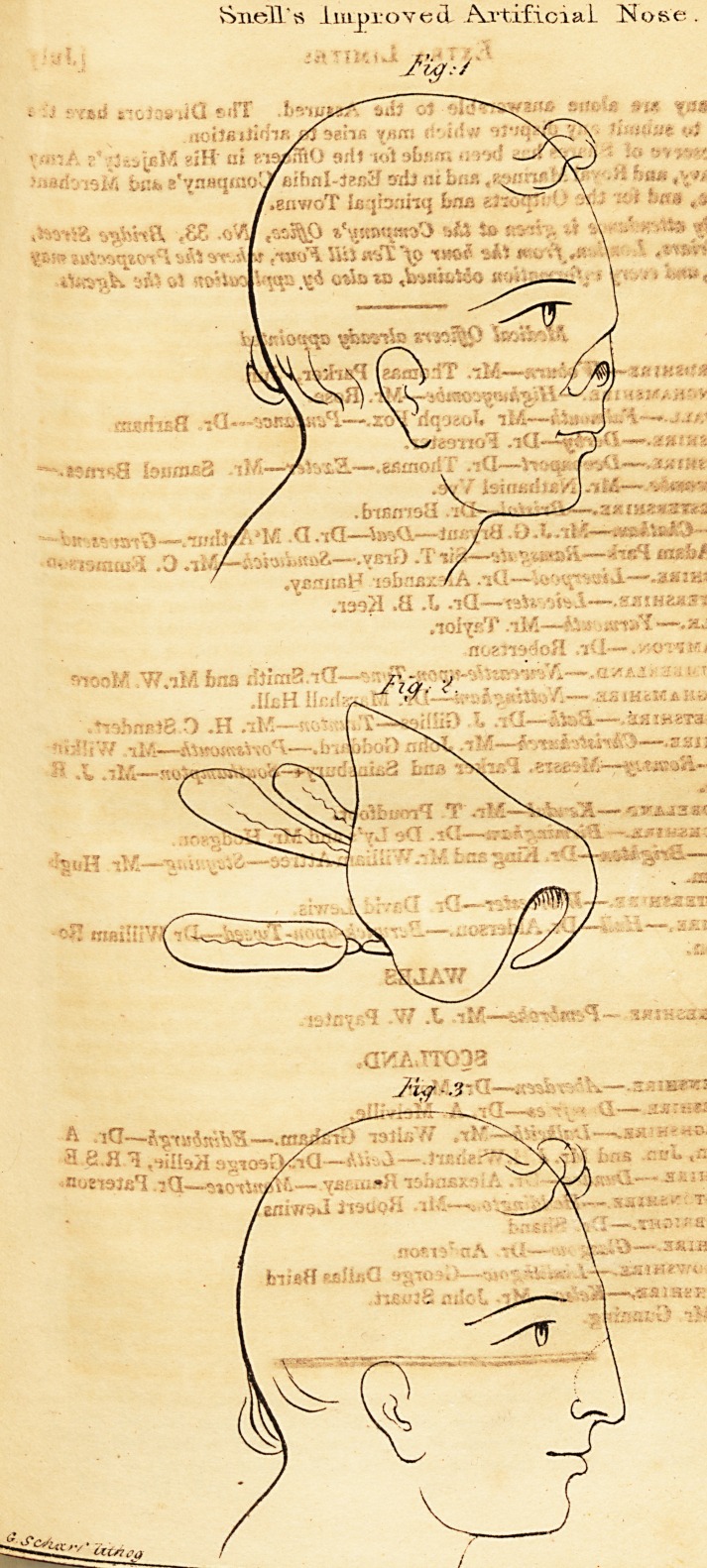# Extra-Limites

**Published:** 1825-07-01

**Authors:** 


### EXTRA-LIMITES.

### I.

### AN IMPROVED ARTIFICIAL NOSE.

By J. Snell, Dentist, Member of the Royal College of Surgeons, &c.

I was applied to in November last, by an officer in the army, who had lost a great part of the nose, and who expressed the greatest aversion to wearing an artificial one made after the manner generally adopted, (with spectacles, or by a spring, passing from the occiput over the head, and down the forehead, to which the nose is attached.) He was anxious to know whether one could not be formed which might be held in apposition with the face, without these external attachments. Having long made that part of mechanical surgery, which has for its object the supplying deficiencies of the mouth and face, my particular study, I conceived it no difficult matter to make an artificial nose which would do away with the unpleasant appearance of the spectacles and head-spring. I accordingly constructed one which was held in close contact with the face, in the following simple manner:—A correct model was first taken of the defective parts, which was cast in brass, and upon which a thin gold plate was accurately fitted, in the manner generally adopted by jewellers. To the inner surface of this plate, at that part which was to form the septum, were soldered three pieces of gold wire, which terminated, each, by a small flat plate, pierced with holes, for the purpose of sewing to its outer surface, a covering of Indian-rubber. These gold wires were rendered highly elastic.

Upon the outer side of the principal plate was next fitted a piece of ivory, so as entirely to cover it; the extreme edges of the ivory being intended to come in close contact with the face. This ivory was then carved to the exact shape and fashion of such a nose as appeared most likely to be suitable for the size and contour of the face for which it was intended—the under part being hollowed out to form the nostrils, rendering it very light and thin. The gold and bone were now rivetted to each other firmly, by small gold pins. The artificial nose was then placed upon the face, and an artist coloured it in oil, exactly to resemble the surrounding parts, both in colour and character.

The nose was held in its position upon the face by the three elastic wires. The two lower ones, having a tendency to press outwards during confinement, pressed against the lateral walls of the nasal cavity. The upper spring having a similar tendency, pressed against the upper roof of the same cavity. The Indian-rubber was used for the purpose of defending the parts from the effects of the pressure of the springs.

The nose might, therefore, be put on and taken off with the greatest facility. The peculiarity of the instrument and the sensibility of the wearer, deprived me of an opportunity of shewing this case to any but the two medical gentlemen who recommended me the patient, and who have since politely honored me with letters, expressive of their favourable opinion of its superiority over those in common use.

Since the above Artificial Nose was constructed, I have made one upon a similar principle, for a medical gentleman, the successful application of which, and the satisfaction expressed by the wearer, confirm what I have before said of its superiority over those in present use.*

111, Crawford Street,
Montagu Square.

(Copy.)

I have had one opportunity of examining an Artificial Nose made by Mr. Snell, and I am of opinion, that his mode of fixing it is decidedly superior to any hitherto adopted.

Charles Street, St. James's, JAMES WARDROP.
April 7th, 1825.

(Copy.) 17, New Burlington Street,
April 4th, 1825.

I have much pleasure in expressing my satisfaction with the manner in which Mr. Snell lately adapted an Artificial Nose in the case of Mr. ——. The objection, on the part of the patient, to the usual concomitants of an artificial nose—a spectacle frame—by means of which the nose is attached to the face, rendered some other mode of connexion necessary. This, Mr. Snell successfully accomplished, and in a manner which, by the *facility of the application of the Artificial Nose, the closeness of its junction to the face, and the firmness of its attachment* thereto, offered more than an equivalent to that by means of a spectacle frame.

J. M. ARNOTT.

---

*Explanation of the Plate.*

*Fig.* 1.—The appearance of the Face before the Artificial Nose was attached.
*Fig.* 2.—The Artificial Nose.
*Fig.* 3.—The appearance of the Face with the Nose attached.

---

### II.

*To the Editor of the Medico-Chirurgical Review.*

8, Broughton Street, Edinburgh,
June 9th, 1825.

SIR,

Permit me to draw the attention of medical men and of the public to this point,—Whether, in *acute hydrocephalus*, when life is despaired of, and all other remedies pronounced useless, recourse ought not to be had to the hand of a skilful surgeon in drawing off the water, either by means of a cannula introduced into the ventricles, or, if the effusion is over the whole surface of the brain, between the arachnoid membrane and the pia mater, by simply puncturing the membranes with a lancet.

---

* I would refer those who may be interested in this case, to Mr. Weiss, Instrument Maker, Strand, who saw the latter gentleman both before and after the nose was supplied.

I find a great difference of opinion among medical men upon this subject; some, for whom I entertain the very highest opinion, going so far as even to disapprove of the attempt to ascertain, by experience, the practical importance of the operation. I judge, from your introductory observations on Mr. Money's case in your last number, page 478, that, as at present advised, you have no great faith in the operation; but, I shall be much disappointed if you are not open to conviction, which can hardly be said of those who discourage the attempt and even forbid it.

I am unfortunately under a melancholy conviction of its utility, and am, therefore, anxious to know what experience may teach us upon this matter. It is possible, that I may have been run away with too much from parental hopes and anxiety in a case, where the existence of the patient was a source of greater happiness to me than all this world can bestow, and therefore, I think it right to give a summing up of the objections and answers that I have heard. It is said:—

*First,*—That the operation of introducing a cannula into the ventricles will produce instant death.

*Answer.*—Mr. Lizars' case in the Edinburgh Journal of April 1821, p. 243, where he introduced it not less than twenty times in the course of three months, and often with benefit, never with injury, together with the cases there referred to,—Dr. Freckleton's case in the same journal, p. 240, Mr. Money's case abovementioned, the experiments of Fleurens, proving how much more liberty can be taken with the brain locally, as distinguished from generally, than was believed, provided the sensorium or origin of the nerves be not injured,—and the numerous accidents from gun-shot wounds, fractures, &c. where portions of the brain are even removed, and one that I am acquainted with, where the head of an arrow was successfully extracted from the brain of a boy ten years of age, prove that the operation can be performed with impunity. See Mr. Wise's observations in the Extra-Limites of your last Number, p. 536.

*2d. Objection.*—That if there is water at the base of the brain as well as in the ventricles, it can not be got out.

*Answer.*—It is not always certain that there is water at the base of the brain; but, in a case where I attended the dissection of the brain of a child that had died of this disease, and where I had recommended the operation and had brought Mr. Lizars to perform it, when the parents changed their mind and would not consent to it, the passage between the base and the ventricles of the brain, discovered by Bichat, was pointed out at my request by Mr. Lizars, as well as the communication by the arachnoid membrane; so that, where there has been no adhesion from disease, water from every part of the brain, and even from the very bottom of the spine, will flow out at the cannula or opening made by the lancet, as the case may require, by the laws of gravitation, and by the influence of respiration on the blood distributed to the brain.

*3d Objection.*—That the operation can be of no use.

*Answer.*—This seems to me, in the present state of our knowledge, to be too violent a presumption. In the *chronic* state of the disease, I fear there is always a derangement of structure or disease in the brain itself, and where that is the case, I agree that the operation would do no good; but, in the *acute hydrocephalus*, produced, as I believe it to be, among children under two years of age, by the irritation of teething, in nine cases out of ten, where a turgescence of the vessels of the brain has terminated in effusion, and where the brain itself is perfectly sound and healthy, what right have we to say that, after safely relieving the brain from the pressure of the water, it is impossible for it to recover the function of absorption, or, in other words, that the balance between the absorbent and exhalent systems can not, by the power of mercury or any other means, be restored to this organ as we know it can be to other organs. In two cases of *acute hydrocephalus*, where I have now witnessed the *post mortem* operation and dissection by Mr. Lizars, the brain was perfectly sound and healthy, and he was of the same opinion that I was; that if the pressure of the water had been removed in life, as it might have been, (in one of the cases by the introduction of the cannula, and in the other, by simply puncturing the membranes with a lancet,) there was a great chance of recovery. Dr. Farquharson, whose abilities are of a very superior order, and who, notwithstanding his great modesty, bids fair to tread in the steps of his late father, long one of the most accomplished physicians of this place, was of the same opinion.

There is considerable authority upon this subject. In the first place, there is that of Hippocrates, that of Mr. John Bell, and that of Dr. Mason Good, upon which I have principally relied, being familiar with his writings from having been the first publicly to recommend his Nosology in this place. I am, moreover, informed by Mr. Dick, whom I had the pleasure and satisfaction of bringing forward as a lecturer, in this town, on veterinary surgery, that, to his knowledge, the operation has been performed with success in sheep. Among breeders of poultry, and particularly of turkies, I understand it is very common.

By the publicity and discussion which has been given to this subject, I trust that, through the means of your wide, and deservedly wide, circulating Review, some valuable information may be communicated to the world.

I have written to a friend at Paris upon the subject, and I wish to invite information publicly or privately; because, after one of these *post mortem* operations by Mr. Lizars, I hazarded an opinion, that the time would come when, in desperate cases of this kind, the operation of drawing off the water would become as common as cutting for the stone is now, to which my old friend and venerable preceptor Dr. Barclay, who was present, added "and much more simple."

The difficulty of establishing this operation must necessarily be greater than that of lithotomy, and you know how long it was before medical men could be brought to approve of that operation.

It neither can nor ought to be attempted but by a skilful surgeon. In nine cases out of ten, the little innocent will be cut off while other remedies are pursuing, and where that does not happen, few parents will give their consent, if the medical man, who enjoys their confidence, withholds his sanction to it, or discourages it in any manner.

In some sudden and severe cases of palsy, where the patient can neither write nor speak, although his intellectual faculties are perfect, and the physician shall be of opinion that the paralytic symptoms are produced entirely by the pressure of water on some part of the brain, (and such a case I have seen) how important may this operation be, if it should do nothing more than restore the faculty of speaking or writing only for a few weeks, days, or even hours, so as to allow the patient time to make his will. Upon the same principle, Sir A. Cooper justifies and recommends the operation of tying the great aorta.

My medical education has been altogether that of a physician; but, I cannot shut my eyes to the benefit mankind is receiving from the brilliant operations in surgery, which have been performed and established in my own day.

I remain, dear Sir, with great respect,

Your faithful Servant,

WILLIAM SCOTT, M. D.

### III.

*The Annual Report of the General Committee of the Associated Apothecaries and Surgeon Apothecaries of England and Wales, received and adopted at the Annual General Meeting of the Association, held at the Crown and Anchor Tavern, Strand, July 7th,* 1824.

JOSEPH HAYES, Esq. *President.*

Your Committee respectfully submit to the consideration of the General Meeting, the following statements and observations.

The instructions of the last General Meeting have been kept in view, as far as circumstances have permitted, and several interesting communications, on the state of the Medical Profession, and on medical and surgical subjects, have been received.

The Volume of Transactions of this Association, published since the last Annual Meeting, has placed on record the labours of the Society for the improvement of the Medical Profession and the advancement of the Public Welfare: this historical account, comprising the entire period since the formation of the Association, necessarily increased the extent of the work, an inconvenience not to be anticipated in any future volume.

Of the prizes offered by the Association for Essays on Medical Education, and on Inflammation, the former only has been awarded, the period for the latter having been extended, under certain limitations, to another year.

The essay to which the premium of the Gold Medal has been adjudged, your Committee trust will be found useful to the Profession, as well as creditable to the zeal and ability of the successful candidate; and your Committee confidently anticipate that the subjects of useful research pointed out by the Association, may call forth the efforts of many whose labours may be beneficial to science and to humanity.

Whilst your Committee has had the gratification of witnessing the very commendable exertions of students to qualify themselves for the duties of an arduous profession, they have to regret that these exertions have been met, not with correspondent liberality, but by restrictive regulations on the part of the College of Surgeons: far from improvement in science being secured by these regulations, the highest attainments are to be set at nought, unless the fees exacted for instruction have passed into certain channels pointed out by the Court of Examiners; your Committee deem it right that the attention of the Profession should be directed to these regulations, for, however pure the motive of the Examiners may have been, the obvious tendency of such restrictions appear to be, to give an undue preference to themselves, their colleagues, and their relations or dependants, to the exclusion of others, not less competent to the task of instructing.

That regulations founded on arbitrary and absurd distinctions cannot now, as formerly, be forced upon the public, may be inferred from the triumph of the Governors of one of the first hospitals in the Metropolis, who have preferred a surgical candidate of their own choosing, disregarding and setting at defiance the trammels by which such elections have too often been fettered.

In addition to the regulations above alluded to, there appears another point deserving the attention of the profession; a technical difficulty has occurred in proving the certificate of qualification, according to the Apothecaries' Act, which places apothecaries residing at a distance from London, who may be necessitated to seek legal redress, in a much worse predicament than those who were in practice at the time of passing the Act; the particulars of which have been ably stated in a recent number of the Medical Repository.

The attention of the Society is also called to certain regulations, which, it appears to the Committee, need some attention: these respect the title of the Association, the election of its officers, and the power to withhold, under certain circumstances, the prizes for the essays.

The object of the Association, in publishing occasional Volumes of Transactions, being, to stimulate to intellectual exertion, to diffuse medical knowledge, and to augment the respectability and usefulness of the general practitioner; it is confidently hoped that gentlemen who duly estimate the value of these objects, will, by communications of an interesting nature, endeavour to promote them.

The Funds of the Association, notwithstanding the premiums and other expences, remain nearly in the same state as last year.

Your Committee cannot close this statement, without allusion to the loss which this Association, and the society at large, have sustained, in the death of Mr. Charles Thomas Haden; devoted to science and philanthropy, his amiable manners endeared him to his associates, whilst his virtues afforded an example worthy of imitation.

*Published by Order of the General Meeting.*

JOHN POWELL, *Sec.*

---

### IV.

### CROWN LIFE ASSURANCE COMPANY.

*Instituted for the Assurance of Lives, the granting of immediate and deferred Annuities, deferred Sums, Endowments for Children, and also for the purchase of Contingent and Reversionary Property. No. 33, Bridge-Street, Blackfriars:*

CAPITAL £1,500,000,

IN 30,000 SHARES OF £50 EACH.

*Directors.*—William Peat Litt, Esq. Chairman.—John Wells, Esq. M. P. Deputy-Chairman—William R. Cosway, Esq.—James Colquhoun, Esq.—James Colvin, Esq.—Captain J.W.D.Dundas,R.N.—James Farquhar, Esq. M. P.—Thomas Harrison, Esq.—George H. Hooper, Esq.—John Kirkland, Esq.—Major Moody, *Royal Engineers.*—Sir Francis Ommaney, M. P.—Thomas Solly, Esq.—Alexander Stewart, Esq.—William Whitmore, Jun. Esq.—John Wilson, Esq.—William Wilson, Esq.

*Auditors.*—John Joseph Harrison, Esq.—Isaac Solly, Jun. Esq.—Henry Stock, Esq.

*Trustees.*—John Wells, Esq. M. P.—James Farquhar, Esq. M.P.—Sir Francis Ommaney, M. P.—George Henry Hooper, Esq.—Alexander Stewart, Esq.

*Bankers.*—Messrs. Whitmore, Wells, and Whitmore, Lombard-street.

*Standing Counsel,*—Charles Ellis, Esq.

*Physician,*—Dr. James Johnson, Physician Extraordinary to H.R.H. the Duke of Clarence, Suffolk-place.

*Surgeon,*—James Wardrop, Esq. F.R.S. Ed. and Surgeon Extraordinary to the King, Charles-street, St. James's.

*Solicitor.*—Thomas Haddan, Esq.—*Actuary,*—Mr. J. M. Rainbow.
*Secretary,*—Mr. T. G. Conyers.

---

The distinguishing feature of this Institution is the extension of the advantages of the system of Life Assurance to classes of individuals who are at present altogether excluded from them, except at premiums quite disproportionate to the actual risk.

It is also intended to combine the ordinary business of Life Assurance upon principles which are the result of the experience of the existing Establishments, and which, whilst they afford adequate security to the Company, will, at the same time, be found to present important advantages to the public.

For the ordinary Risks of Life Assurance an entirely new set of Tables has been formed for this Company, in calculating which great pains have been taken to apportion the Rates to the risk incurred at the several ages, and the Scale now presented to the Public will be found on inspection to afford liberal terms to the Assured.

Two Thirds of such Profits as shall periodically be declared divisible will be apportioned among Assurers for the whole term of life, and may be applied to the reduction of the future annual Premiums, or to the increase of the sum assured, as may be desired. The remaining one Third of the Profits will be apportioned among the Shareholders according to the Deed of Settlement, and thus persons being Shareholders and effecting Insurances for the whole Term of Life become entitled to participate in the whole of the Profits.

Permission is granted (without any additional charge) to persons insured by this Company, to proceed by Sea, in decked vessels or steam-boats, from one part of the United Kingdom of Great Britain and Ireland to another, and to and from the Islands of Guernsey, Jersey, Alderney, Sark, and Man, and also in time of Peace to and from British Ports, and any foreign Ports between the Elbe and Brest, both inclusive.

The Purchase of their Policies is effected by this Company upon fair and equitable principles.

The Company has also for its object the Insurance of the Lives of Officers in His Majesty's Army, Navy, and Royal Marines (both at home and *upon actual service)*, in the Military and Maritime Service of the Honorable the East-India Company, and in the Merchant Service of the United Kingdom, and the insurance of the lives of individuals *intending to reside abroad*, either permanently or for shorter periods, and of persons *proceeding upon single voyages* to any quarter of the globe, or *remaining at sea*, at premiums proportionate to the actual risk, and calculated upon unquestionable and ascertained data.

This Company will also grant Life Annuities, present and deferred, and Endowments for Children, purchase Life and Reversionary Interests, advance money on Mortgage, or other security, and transact generally all business appertaining to a Life-Assurance Company.

No Charge is made for Admission Fees or Entrance-money, or for Policies, beyond the amount of the Stamp.

Shareholders in this Company are required to insure and keep on foot Insurances to the amount of one half of their Shares, to purchase or sell an Annuity the consideration for which shall be equal to that amount, or to pay an annual fine of 2*s.* 6*d. per* Share upon such moiety. No Proprietor is to be answerable for any claims or demands against the Company beyond the amount of his Share in the subscribed Capital, and the funds of the

Company are alone answerable to the Assured. The Directors have the power to submit any dispute which may arise to arbitration.

A reserve of Shares has been made for the Officers in His Majesty's Army and Navy, and Royal Marines, and in the East-India Company's and Merchant Service, and for the Outports and principal Towns.

*Daily attendance is given at the Company's Office, No. 33, Bridge Street, Blackfriars, London, from the hour of Ten till Four, where the Prospectus may be had, and every information obtained, as also by application to the Agents.*

---

*Medical Officers already appointed.*

BEDFORDSHIRE—*Woburn*—Mr. Thomas Parker, Jun.
BUCKINGHAMSHIRE.—*Highwycombe*—Mr. Rose.
CORNWALL.—*Falmouth*—Mr Joseph Fox.—*Penzance*---Dr. Barham.
DERBYSHIRE.—*Derby*—Dr. Forrester.
DEVONSHIRE.—*Devonport*—Dr. Thomas.—*Exeter*—Mr. Samuel Barnes.—*Ilfracombe*.—Mr. Nathaniel Vye.
GLOUCESTERSHIRE.—*Bristol*—Dr. Bernard.
KENT.—*Chatham*—Mr. J. G. Bryant—*Deal*—Dr. D. M'Arthur.—*Gravesend*—Mr. Adam Park—*Ramsgate*—Sir T. Gray.—*Sandwich*—Mr. C. Emmerson.
LANCASHIRE.—*Liverpool*—Dr. Alexander Hannay.
LEICESTERSHIRE.—*Leicester*—Dr. J. B. Keer.
NORFOLK.—*Yarmouth*—Mr. Taylor.
NORTHAMPTON.—Dr. Robertson.
NORTHUMBERLAND.—*Newcastle-upon-Tyne*—Dr. Smith and Mr. W. Moore.
NOTTINGHAMSHIRE.—*Nottingham*—Dr. Marshall Hall.
SOMERSETSHIRE.—*Bath*—Dr. J. Gillies—*Taunton*—Mr. H. C. Standert.
HAMPSHIRE.—*Christchurch*—Mr. John Goddard.—*Portsmouth*—Mr. Wilkinson.---*Romsey*—Messrs. Parker and Sainsbury.—*Southampton*—Mr. J. R. Keele.
WESTMORELAND.—*Kendal*—Mr. T. Proudfoot.
WARWICKSHIRE.—*Birmingham*—Dr. De Ly's and Mr. Hodgson.
SUSSEX.—*Brighton*—Dr. King and Mr. William Attree—*Steyning*—Mr. Hugh Ingram.
WORCESTERSHIRE.—*Worcester*—Dr. David Lewis.
YORKSHIRE.—*Hull*—Dr. Alderson.—*Berwick-upon-Tweed*—Dr. William Robertson.

### WALES.

PEMBROKESHIRE.—*Pembroke*—Mr. J. W. Paynter.

### SCOTLAND.

ABERDEENSHIRE.—*Aberdeen*—Dr. Moir.
DUMFRIESHIRE.—*Dumfries*—Dr. A. Melville.
EDINBURGHSHIRE.—*Dalkeith*—Mr. Walter Graham.—*Edinburgh*—Dr. A. Duncan, Jun. and Mr. J. H. Wishart.—*Leith*—Dr. George Kellie, F.R.S.E.
FORFARSHIRE.—*Dundee*—Dr. Alexander Ramsay.—*Montrose*—Dr. Paterson.
HADDINGTONSHIRE.—*Haddington*—Mr. Robert Lewins.
KIRKCUDBRIGHT.—Dr. Shand.
LANARKSHIRE.—*Glasgow*—Dr. Anderson.
LINLITHGOWSHIRE.—*Linlithgow*—George Dallas Baird.
ROXBURGHSHIRE.—*Kelso*—Mr. John Stuart.
PARIS.---Mr. Gunning.

## Figures and Tables

**Fig. 1 Fig. 2. Fig 3 f1:**